# Association between interleukin-6, C-reactive protein and adiponectin with adiposity: Findings from the 1993 pelotas (Brazil) birth cohort at 18 and 22 years

**DOI:** 10.1016/j.cyto.2018.04.020

**Published:** 2018-10

**Authors:** Ana Maria Baptista Menezes, Paula Duarte Oliveira, Fernando César Wehrmeister, Helen Gonçalves, Maria Cecilia F. Assunção, Luciana Tovo-Rodrigues, Gustavo Dias Ferreira, Isabel O. Oliveira

**Affiliations:** a Federal University of Pelotas – Postgraduate Program in Epidemiology, Rua Marechal Deodoro, 1160, 3° andar, Pelotas, RS. Zip code: 96020-220, Brazil; b Federal University of Pelotas, Department of Physiology and Pharmacology Campus Capão do Leão, s/n. Pelotas, RS. Zip code: 96010-900, Brazil

**Keywords:** Interleukin-6, Adiponectin, C-reactive protein, Obesity, Adiposity, Inflammatory markers, Cohort studies, BIA, bioelectrical impedance analysis, BMI, body mass index, CRP, C-reactive protein, DXA, dual-energy X-ray absorptiometry, FM, fat mass, FMI, fat mass index, SD, standard deviation, SE, standard error, WC, waist circumference

## Abstract

**Background:**

Most of the literature has shown a direct association between adiposity, interleukin-6 (IL-6) and C-reactive protein (CRP), and an inverse association with adiponectin. The aim of this paper was to assess the association of obesity measurements and IL-6, CRP and adiponectin.

**Methods:**

We performed cross-sectional/longitudinal analysis in the 1993 Birth Cohort in Brazil. Associations between IL-6 (pg/mL), CRP (mg/L) and adiponectin (µg/Ml) with body mass index (BMI), waist circumference (WC), percentage total fat mass (FM) (BODPOD) and trunk FM (DXA) were verified. Four trajectory variables were generated: (a) highest tertile/obesity according to BMI in both follow-ups; (b) highest tertile/obesity only at 18 years; (c) highest tertile/obesity only at 22 years; (d) not in the highest tertile/not obese at both ages.

**Results:**

We found a direct association for IL-6 and CRP and an inverse association for adiponectin with measures of adiposity. For instance, for females, the highest mean IL-6 and CRP, respectively, was for those who had been in the obese category at 18–22 years (2.49; 3.75), in the highest tertile of WC (2.11; 3.08), in the highest % of FM (1.56; 3.30 l), and in the highest tertile of trunk FM (2.07; 3.26) (p < 0.001 for all these results in the adjusted analysis). Adiponectin showed an inverse association for the above variables, with lower mean values for males than females.

**Conclusion:**

There was a longitudinal and direct association between adiposity and IL-6 and CRP, and an inverse association with adiponectin at 22 years.

## Highlights

• There is a positive association between IL and 6 and CRP with body adiposity.
• The analysis between adiponectin and adiposity shows an inverse association.
• These associations are both cross-sectional and prospective and cumulative.
• Women show higher mean IL-6, CRP and adiponectin when compared with men.
• The associations were found in a cohort at 18 and 22 years of age and in both sexes.

## 1 Introduction

Obesity during childhood and adolescence has been associated with several related morbidities in adult life, such as diabetes, coronary heart disease and some types of cancer [1]. It has been suggested that obesity is a chronic inflammatory condition [2], with increased production of cytokines [3]. Circulating levels of inflammatory markers such as C-reactive protein (CRP), interleukin-6 (IL-6), interleukin-1 receptor antagonist (IL-1RA), and tumor necrosis factor-a (TNF-a) have been reported in younger to older adults to be positively associated with increased adiposity [4], [5], [6]. There is also some evidence that IL-6 and CRP are predictors of mortality [7], [8], [9]. In contrast to most adipokines, adiponectin is a 244-aminoacid long polypeptide and its plasma concentration is lower in obese individuals [10], [11] and according to some studies, adiponectin can be protector of mortality, opposed to IL-6 and CRP [12], [13], [14]. However, there are conflicting results in the literature for adiponectin [15]. An increase of 1 mg/mL in adiponectin concentration has been associated with either a decreased or an increased risk for cardiovascular events in chronic kidney disease patients [12], [16], [17]. More detailed studies have shown that is fat quality, and not fat mass (FM), that is the driving force for adiponectin expression [18].

The effects of different obesity markers such as body mass index (BMI), waist circumference (WC), % fat mass index (FMI) on cytokine levels have been poorly investigated in Latin America. In a Swiss population-based sample aged 35–75 years (CoLaus Study) [19], it was found that participants with high percentage of body fat or abdominal obesity measured by bioelectrical impedance analysis (BIA) had higher IL-6 levels, but no association between IL-6 levels and obesity markers was found on multivariate regression.

Due to the ongoing epidemic of obesity in Brazil [20], the objective of this study was to assess in a cross-sectional and in a prospective way the association of different obesity measurements (BMI, WC and % FM) and IL-6, CRP and adiponectin at 22 years, in a population Birth Cohort in Southern Brazil.

## 2 Methods

In 1993, all hospitals in the city of Pelotas were monitored daily, and mothers of newborns were invited to participate in a prospective study. From the 5265 live births in the city, 5249 were enrolled in our birth cohort study. Mothers were interviewed shortly after delivery on demographic, socioeconomic, and health-related variables and newborns were weighted and measured by the study team [21].

Subsamples of the cohort were followed up during childhood [22], [23], [24] and all cohort members were sought when they had reached the mean age of 11, 15, 18 and 22 years [25], [26], [27].

At 18 and 22 year follow-up visits, non-fasting blood samples were drawn by venipuncture using vacutainer tubes. All samples were processed in the laboratory, stored in freezers at ultra-low temperature in the same place and registered in a central biorepository. IL-6 was measured by the Quantikine® HS Human IL-6 immunoassay kit (R&D Systems®, Inc.; Minneapolis, MN55413, USA); CRP was measured by immunoturdimetric assay (Labtest Diagnóstica SA, Minas Gerais, Brazil) and adiponectin was assayed with the ELISA Quantikine Human Total Adiponectin Immunoassay kit (R&D Systems, Inc., Minneapolis, USA); intra-assay and inter-assay coefficients of variation were 9.1% and 13.2%, respectively.

The present analysis includes all participants who had measurements of IL-6, CRP and/or adiponectin at 18 and/or 22 years; IL-6 and CRP have been transformed in logarithm due to its asymmetric distribution and the results reported as exponential mean with its standard error (SE); adiponectin had a normal distribution and was analyzed in its original unit and reported as mean and SE. The exclusion criteria for the blood sample were refusal for collecting blood and pregnancy in women.

The main exposure variables for the present paper, collected at 18 and 22 year follow-ups, were BMI (kg/m^2^), waist circumference (WC; measured in cm by trained personal using a tape measure in the narrowest point of the abdomen), % of total FM measured by the BOD POD (BOD POD® Composition System; COSMED, Albano Laziale, Italy) [28] and % of segmented (trunk) FM measured by DXA (model Lunar Prodigy Advance®; GE Healthcare, Freiburg, Germany).

BMI was categorized as underweight/normal (≤24.9 kg/m^2^), overweight (25.0–29.9 kg/m^2^) and obese (≥30 kg/m^2^) [29], and WC and % FM (BOD POD and trunk by DXA) as tertiles. Also, four variables were generated classifying individuals according to BMI, WC, total FM and trunk FM as follows: in the highest tertile or obese according to BMI at 18 and 22 years, in the highest tertile or obese only at 18 years, in the highest tertile or obese only at 22 years and not in the highest tertile or not obese in both (reference category).

Interviewers underwent standardization testing before beginning of field work and every two months afterward to determine repeatability and validity of weight, height and waist circumference measurements.

The other co-variables were skin color (white, black and other), schooling (complete years), asset index (in quintiles), smoking (never, former and current), harmful alcohol intake (Alcohol Use Disorders Identification Test – AUDIT ≥ 8 points [30], physical activity measured through a standardized and previously tested questionnaire [31] (categorized as inactive < 300/150 min a week and active for ≥ 300/150 min a week, according to cut-off points for adolescent and adults, at 18 and 22 years, respectively) and medical diagnosis at any time during life referred by the participants (yes or no for hypertension, asthma, diabetes), at 18 and at 22 years.

The sample characteristics were described using absolute and relative frequencies for categorical variables and means and SD for continuous variables. Crude and adjusted linear regressions for the above co-variates have been conducted. All analyses were sex-stratified due to the significant interaction for body composition and sex (p < 0.001) and were performed using software Stata version 12.2 (Stata Corp., College Station, TX, USA). The results were reported in pg/mL and mg/L for IL-6 and CRP, respectively, in means and SE after exponential of the logarithm result. For adiponectin the results were reported in µg/mL. Values of p < 0.05 in the Wald test for linear regression or heterogeneity were considered statistically significant.

All cohort follow-ups projects were approved by the Federal University of Pelotas Ethics Committee. The follow-ups that were used in the present study were approved under protocols 05/11 and 1.250.366 for the 1993 18 and 22 year follow-ups, respectively. The cohort participants, or their caregivers, signed the term of free and informed consent prior to participation.

## 3 Results

The response rate of the 1993 Birth Cohort at 18 and 22 years of age was 81.4% and 76.3%, respectively. The total sample size for the present analyses was 3877 and 3483 for the 18 and the 22 year evaluations, respectively.

Table 1 describes the characteristics of the sample, classified by sex according to each follow-up visit. Most of them were white, and nearly half of them had 9–11 years of schooling. Men were wealthiest, smoked more, showed a higher harmful alcohol intake and were more physically active compared to women in both visits. No sex differences were found in the prevalence of morbidities according to medical diagnosis; the point prevalence varied from 3.3 for diabetes among women to around 23% of asthma among men.

**Table 1:** Description of the sample (individuals with IL-6, CRP and/or adiponectin information) according to demographic, socioeconomic, behavioral and health variables, stratified by sex, at 18 (n = 3877) and 22 years old (n = 3483).

	18 years		22 years	
	Males (n = 1938) N (%)	Females (n = 1939) N (%)	Males (n = 1540) N (%)	Females (n = 1598) N (%)
*Skin color*				
White	1175 (64.3)	1202 (63.2)	992 (63.6)	1089 (62.7)
Black	271 (14.8)	273 (14.4)	232 (14.9)	270 (15.5)
Others	381 (20.9)	426 (22.4)	336 (21.5)	379 (21.8)

*Schooling (complete years)*				
0–4	127 (6.6)	55 (2.8)	70 (4.2)	28 (1.5)
5–8	906 (46.9)	669 (34.6)	544 (32.9)	395 (21.7)
9–11	841 (43.5)	1126 (58.2)	659 (39.8)	773 (42.5)
≥12	59 (3.0)	86 (4.4)	382 (23.1)	623 (34.3)

*Asset index (quintiles)*				
1st	313 (16.2)	464 (24.0)	271 (16.3)	428 (23.3)
2nd	391 (20.2)	373 (19.3)	304 (18.3)	386 (21.3)
3rd	388 (20.1)	394 (20.4)	340 (20.5)	365 (20.1)
4th	426 (22.0)	361 (19.7)	369 (22.2)	328 (18.2)
5th	415 (21.5)	344 (17.8)	375 (22.6)	310 (17.1)

*Smoking status*				
Never smoker	1493 (77.2)	1512 (78.1)	1149 (69.2)	1385 (76.2)
Former	142 (7.3)	167 (8.6)	167 (10.1)	184 (10.1)
Current smoker	299 (15.5)	257 (13.3)	344 (20.7)	248 (13.7)
*Harmful alcohol intake (AUDIT ≥ 8 points)*	693 (35.8)	340 (17.6)	501 (30.2)	260 (14.3)

Total physical activity*				
Inactive	493 (25.6)	1002 (51.8)	421 (25.4)	762 (42.0)
Active	1436 (74.4)	931 (48.2)	1234 (74.6)	1054 (58.0)

*Medical diagnosis*				
Hypertension	161 (8.3)	187 (9.7)	149 (9.0)	172 (9.5)
Diabetes	86 (4.5)	65 (3.4)	68 (4.1)	83 (4.6)
Asthma	444 (23.0)	384 (19.9)	386 (23.3)	397 (21.9)

* Classified as inactive those who do not reach 300 and 150 min/week of physical activities in leisure and commuting, at 18 and 22 years, respectively. AUDIT: Alcohol Use Disorder Identification Test.

Weight, BMI, WC, % fat mass according to BOD POD and in trunk according to DXA were higher at 22 than at 18 years for both sexes. The mean IL-6 and CRP at 22 years, respectively, were higher among women (1.80 pg/mL and 3.93 mg/L) than in men (1.60 pg/mL and 1.84 mg/L) (Table 2).

**Table 2:** Description of the sample (individuals with IL-6, CRP and/or adiponectin information) according to nutritional status, body composition and IL-6 at 18 (n = 3877) and 22 years old (n = 3483), stratified by sex.

	Males Mean (SD)		Females Mean (SD)	
	18 years	22 years	18 years	22 years
Weight (kg)	70.7 (14.2)	76.2 (16.1)	61.0 (13.9)	66.1 (15.4)
Height (cm)	173.8 (7.0)	174.4 (7.1)	161.0 (6.5)	161.1 (6.6)
BMI (kg/m^2^)	23.4 (4.2)	25.1 (4.8)	23.5 (4.7)	25.6 (5.7)
Waist circumference (cm)	78.5 (9.6)	82.9 (10.8)	73.8 (9.7)	77.3 (11.7)
Fat mass (% – BOD POD)	16.7 (8.9)	20.8 (9.9)	32.7 (7.8)	35.8 (8.5)
Trunk fat mass (% – DXA)	18.8 (10.6)	24.2 (11.5)	36.0 (9.3)	39.4 (9.8)
IL-6 (pg/mL)*	1.61 (1.95)	1.60 (1.87)	1.78 (1.95)	1.80 (1.80)
IL-6 (log pg/mL)*	0.21 (0.64)	0.19 (0.66)	0.30 (0.67)	0.32 (0.68)
CRP (mg/L)**	1.52 (2.93)	1.84 (4.92)	3.15 (4.72)	3.93 (8.45)
CRP (log mg/L)**	−0.40 (1.18)	−0.34 (1.22)	0.30 (1.35)	0.48 (1.32)
Adiponectin (µg/mL)***	14.26 (7.04)	7.97 (3.98)	17.04 (7.91)	10.72 (4.72)

BMI: body mass index. IL-6: interleukin-6; CRP: C – reactive protein.

* N 18 years: 1545 males/1613 females; 22 years: 1540 males/1598 females.

** N 18 years: 1933 males/1936 females; 22 years: 1659 males/1816 females.

*** N 18 years subsample: 138 males/137 females; 22 years: 1660 males/1822 females.

Crude and adjusted regression cross-sectional analysis for IL-6, CRP and adiponectin in males and females, according to three categories of IMC (low/normal, overweight and obese) and tertiles of WC, % of fat mass by BOD POD and % fat mass in the trunk by DXA are shown in Tables 3a and 3b. It can be observed a highly significant direct association of IL-6 and CRP at 18 and at 22 years with all measurements of adiposity at each age and a dose-response pattern in both crude and adjusted models in both sexes (Table 3a, Table 3b). Since the results in the adjusted analysis are quite similar to the crude ones, the data pointed out here it will be the one referred to the adjusted values. On the contrary, adiponectin in both sexes and ages showed an inverse association with all measurements of obesity and adiposity.

**Table 3a:** Crude and adjusted linear regressions between nutritional status/body composition and IL-6, CRP and adiponectin at 18 and 22 years old, males.

	18 years						22 years					
	IL-6 (pg/mL) N = 1545 Mean (SE)		CRP (mg/L) N = 1933 Mean (SE)		Adiponectin (µg/mL) N = 138 Mean (SE)		IL-6 (pg/mL) N = 1540 Mean (SE)		CRP (mg/L) N = 1659 Mean (SE)		Adiponectin (µg/mL) N = 1660 Mean (SE)	
	Crude	Adjusted	Crude	Adjusted	Crude	Adjusted	Crude	Adjusted	Crude	Adjusted		
BMI	*p < 0.001*	*p < 0.001*	*p < 0.001*	*p < 0.001*	*p = 0.002*	*p = 0.003*	*p < 0.001*	*p < 0.001*	*p < 0.001*	*p < 0.001*	*p < 0.001*	*p < 0.001*
Underweight/normal	1.17 (1.01)	1.17 (1.02)	0.57 (1.02)	0.58 (1.03)	16.29 (0.82)	16.07 (0.82)	1.08 (1.02)	1.08 (1.02)	0.55 (1.03)	0.55 (1.04)	8.61 (0.13)	8.62 (0.13)
Overweight	1.28 (1.03)	1.29 (1.04)	0.87 (1.05)	0.88 (1.06)	10.63 (2.6)	9.52 (2.83)	1.20 (1.02)	1.21 (1.03)	0.77 (1.04)	0.78 (1.05)	7.59 (0.17)	7.49 (0.18)
Obesity	1.95 (1.04)	1.96 (1.06)	1.73 (1.06)	1.80 (1.10)	12.40 (0.86)	12.29 (0.88)	1.84 (1.03)	1.83 (1.04)	1.54 (1.05)	1.49 (1.08)	6.19 (0.26)	6.18 (0.26)

Waist circumference (tertiles)	*p < 0.001*	*p < 0.001*	*p < 0.001*	*p < 0.001*	*p = 0.002*	*p = 0.013*	*p < 0.001*	*p < 0.001*	*p < 0.001*	*p < 0.001*	*p < 0.001*	*p < 0.001*
1st	1.16 (1.03)	1.16 (1.03)	0.52 (1.05)	0.53 (1.05)	15.96 (1.03)	15.67 (1.04)	1.07 (1.03)	1.05 (1.03)	0.50 (1.05)	0.49 (1.05)	8.97 (0.16)	8.99 (0.17)
2nd	1.17 (1.03)	1.18 (1.03)	0.57 (1.05)	0.58 (1.05)	17.13 (1.38)	16.95 (1.43)	1.12 (1.03)	1.14 (1.03)	0.66 (1.05)	0.68 (1.05)	8.06 (0.17)	8.05 (0.17)
3rd	1.38 (1.03)	1.39 (1.03)	1.01 (1.04)	1.04 (1.05)	12.25 (0.80)	12.11 (0.83)	1.45 (1.03)	1.46 (1.03)	1.08 (1.05)	1.08 (1.05)	6.82 (0.17)	6.74 (0.17)

% total fat mass BOD POD (tertiles)	*p < 0.001*	*p < 0.001*	*p < 0.001*	*p < 0.001*	*p = 0.003*	*p = 0.141*	*p < 0.001*	*p < 0.001*	*p < 0.001*	*p < 0.001*	*p < 0.001*	*p < 0.001*
1st	1.14 (1.03)	1.12 (1.03)	0.51 (1.05)	0.51 (1.05)	14.19 (1.10)	14.28 (1.11)	1.09 (1.03)	1.08 (1.03)	0.51 (1.05)	0.49 (1.05)	8.77 (0.17)	8.86 (0.17)
2nd	1.17 (1.03)	1.18 (1.03)	0.60 (1.05)	0.61 (1.05)	17.94 (1.26)	17.37 (1.30)	1.09 (1.03)	1.11 (1.03)	0.60 (1.05)	0.62 (1.05)	8.15 (0.16)	8.10 (0.17)
3rd	1.40 (1.03)	1.43 (1.03)	0.99 (1.04)	1.03 (1.05)	12.78 (0.81)	12.63 (0.84)	1.46 (1.03)	1.48 (1.03)	1.16 (1.05)	1.17 (1.05)	6.94 (0.17)	6.79 (0.18)

% trunk fat mass DXA (tertiles)	*p < 0.001*	*p < 0.001*	*p < 0.001*	*p < 0.001*	*p = 0.054*	*p = 0.051*	*p < 0.001*	*p < 0.001*	*p < 0.001*	*p < 0.001*	*p < 0.001*	*p < 0.001*
1st	1.13 (1.03)	1.12 (1.03)	0.49 (1.05)	0.49 (1.05)	15.73 (1.12)	15.48 (1.20)	1.06 (1.03)	1.03 (1.03)	0.51 (1.06)	0.49 (1.05)	8.91 (0.17)	8.95 (0.18)
2nd	1.16 (1.03)	1.18 (1.03)	0.61 (1.05)	0.63 (1.05)	16.22 (1.34)	16.03 (1.42)	1.08 (1.03)	1.10 (1.03)	0.58 (1.05)	0.61 (1.05)	7.97 (0.17)	7.94 (0.18)
3rd	1.38 (1.03)	1.41 (1.03)	0.95 (1.04)	1.00 (1.05)	12.91 (0.90)	12.73 (0.95)	1.39 (1.03)	1.41 (1.03)	1.07 (1.05)	1.08 (1.05)	7.02 (0.18)	6.86 (0.18)

Regressions performed with IL-6 and CRP on logarithmic scale – results presented in exponential means. P-value by the Wald’s test for linear tendency.

Adjusted for skin color, schooling, asset index, smoking status, harmful alcohol use, physical activity (minutes per week) and medical diagnosis of asthma, diabetes and hypertension.

**Table 3b:** Crude and adjusted linear regressions between nutritional status/body composition and IL-6, CRP and adiponectin at 18 and 22 years old, females.

	18 years						22 years					
	IL-6 (pg/mL) N = 1613 Mean (SE)		CRP (mg/L) N = 1936 Mean (SE)		Adiponectin (µg/mL) N = 137 Mean (SE)		IL-6 (pg/mL) N = 1598 Mean (SE)		CRP (mg/L) N = 1816 Mean (SE)		Adiponectin (µg/mL) N = 1822 Mean (SE)	
	Crude	Adjusted	Crude	Adjusted	Crude	Adjusted	Crude	Adjusted	Crude	Adjusted		
BMI	*p < 0.001*	*p < 0.001*	*p < 0.001*	*p < 0.001*	*p = 0.002*	*p = 0.010*	*p < 0.001*	*p < 0.001*	*p < 0.001*	*p < 0.001*	*p < 0.001*	*p < 0.001*
Underweight/normal	1.19 (1.01)	1.20 (1.02)	1.06 (1.02)	1.07 (1.04)	19.26 (0.92)	18.74 (0.96)	1.10 (1.02)	1.11 (1.02)	1.14 (1.03)	1.11 (1.04)	11.93 (0.14)	11.80 (0.15)
Overweight	1.62 (1.03)	1.61 (1.04)	1.99 (1.05)	2.00 (1.07)	17.46 (3.11)	16.37 (3.15)	1.45 (1.02)	1.44 (1.03)	1.80 (1.04)	1.78 (1.06)	9.93 (0.21)	9.86 (0.21)
Obesity	2.28 (1.04)	2.23 (1.05)	3.77 (1.06)	3.77 (.110)	14.53 (0.97)	15.05 (1.00)	2.50 (1.03)	2.44 (1.04)	3.89 (1.05)	3.88 (1.07)	8.25 (0.24)	8.44 (0.25)

Waist circumference (tertiles)	*p < 0.001*	*p < 0.001*	*p < 0.001*	*p < 0.001*	*p = 0.003*	*p = 0.007*	*p < 0.001*	*p < 0.001*	*p < 0.001*	*p < 0.001*	*p < 0.001*	*p < 0.001*
1st	1.16 (1.03)	1.17 (1.03)	0.89 (1.05)	0.91 (1.05)	20.41 (1.25)	19.87 (1.30)	1.01 (1.03)	1.03 (1.03)	1.01 (1.05)	0.98 (1.05)	12.58 (0.18)	12.48 (0.18)
2nd	1.23 (1.03)	1.24 (1.03)	1.22 (1.05)	1.23 (1.05)	17.63 (1.42)	17.18 (1.46)	1.27 (1.03)	1.26 (1.03)	1.41 (1.05)	1.38 (1.05)	10.95 (0.18)	10.76 (0.19)
3rd	1.73 (1.03)	1.72 (1.03)	2.29 (1.05)	2.31 (1.05)	15.04 (0.91)	15.39 (0.93)	2.05 (1.03)	2.02 (1.03)	2.99 (1.05)	3.02 (1.05)	8.63 (0.18)	8.72 (0.19)

% total fat mass BOD POD (tertiles)	*p < 0.001*	*p < 0.001*	*p < 0.001*	*p < 0.001*	*p = 0.007*	*p = 0.014*	*p < 0.001*	*p < 0.001*	*p < 0.001*	*p < 0.001*	*p < 0.001*	*p < 0.001*
1st	1.17 (1.03)	1.17 (1.03)	0.79 (1.05)	0.80 (1.05)	19.95 (1.34)	19.36 (1.41)	1.02 (1.03)	1.02 (1.03)	0.91 (1.05)	0.90 (1.05)	12.15 (0.18)	12.10 (0.18)
2nd	1.21 (1.03)	1.22 (1.03)	1.32 (1.05)	1.35 (1.05)	18.58 (1.45)	18.46 (1.53)	1.29 (1.03)	1.30 (1.03)	1.55 (1.05)	1.53 (1.05)	10.79 (0.19)	10.57 (0.20)
3rd	1.75 (1.03)	1.75 (1.03)	2.40 (1.05)	2.42 (1.05)	15.21 (0.88)	15.39 (0.90)	2.02 (1.03)	1.99 (1.03)	3.16 (1.05)	3.11 (1.05)	9.14 (0.19)	9.20 (0.19)

% trunk fat mass DXA (tertiles)	*p < 0.001*	*p < 0.001*	*p < 0.001*	*p < 0.001*	*p = 0.034*	*p = 0.074*	*p < 0.001*	*p < 0.001*	*p < 0.001*	*p < 0.001*	*p < 0.001*	*p < 0.001*
1st	1.16 (1.03)	1.15 (1.03)	0.83 (1.05)	0.84 (1.05)	19.74 (1.30)	19.16 (1.36)	1.02 (1.03)	1.03 (1.03)	0.95 (1.05)	0.95 (1.05)	12.47 (0.19)	12.34 (0.19)
2nd	1.22 (1.03)	1.24 (1.03)	1.23 (1.05)	1.26 (1.05)	18.83 (1.59)	18.58 (1.63)	1.25 (1.03)	1.25 (1.03)	1.44 (1.05)	1.40 (1.05)	10.71 (0.19)	10.55 (0.19)
3rd	1.69 (1.03)	1.70 (1.03)	2.29 (1.05)	2.31 (1.05)	15.79 (0.95)	15.99 (0.96)	1.99 (1.03)	1.97 (1.03)	3.01 (1.05)	3.00 (1.05)	8.99 (0.19)	9.09 (0.19)

Regressions performed with IL-6 and CRP on logarithmic scale – results presented in exponential means. P-value by the Wald’s test for linear tendency.

Adjusted for skin color, schooling, asset index, smoking status, harmful alcohol use, physical activity (minutes per week) and medical diagnosis of asthma, diabetes and hypertension.

Tables 4a (males) and 4b (females) shows the longitudinal analysis of the exposures from 18 to 22 years and the outcomes at 22 years old. According to BMI, the highest increase in the mean IL-6 and CRP were for those who were obese at the two follow ups, with a mean IL-6 and CRP of 1.96 (1.07) and 1.55 (1.13) in males (Table 4a) and 2.49 (1.05) and 3.75 (1.11) in females (Table 4b), respectively. The second BMI category with the highest mean IL-6 and CRP was for those who were non-obese at 18 years and became obese at 22 years for both sexes (Table 4a, Table 4b), except for CRP and WC in males, where the mean value for this category was higher than for those obese at both ages (1.12 versus 1.09). The prevalence of obese subjects at both visits was 6.5% for men and 9.4% for women.

**Table 4a:** Crude and adjusted linear regressions between nutritional status/body composition from 18 to 22 years and IL-6, CRP and adiponectin at 22 years, males.

	Prevalence N (%)	IL-6 (pg/mL) N = 1540 Mean (SE)		CRP (mg/L) N = 1659 Mean (SE)		Adiponectin (µg/mL) N = 1660 Mean (SE)	
		Crude	Adjusted	Crude	Adjusted	Crude	Adjusted
BMI ≥ 30 kg/m^2^ at 18 and 22 years		*p < 0.001*	*p < 0.001*	*p < 0.001*	*p < 0.001*	*p < 0.001*	*p < 0.001*
No/no	1290 (84.3)	1.12 (1.01)	1.13 (1.02)	0.62 (1.03)	0.62 (1.03)	8.14 (0.11)	8.12 (0.11)
No/yes	117 (7.6)	1.71 (1.04)	1.72 (1.06)	1.48 (1.07)	1.46 (1.11)	6.33 (0.34)	6.34 (0.35)
Yes/no	25 (1.6)	1.04 (1.11)	1.05 (1.14)	0.79 (1.21)	0.87 (1.26)	9.46 (0.74)	9.44 (0.77)
Yes/yes	99 (6.5)	2.00 (1.04)	1.96 (1.07)	1.61 (1.07)	1.55 (1.13)	6.01 (0.39)	6.01 (0.40)

Waist circumference in the highest tertile at 18 and 22 years		*p < 0.001*	*p < 0.001*	*p < 0.001*	*p < 0.001*	*p < 0.001*	*p < 0.001*
No/no	902 (58.8)	1.10 (1.02)	1.10 (1.02)	0.56 (1.04)	0.56 (1.04)	8.37 (0.13)	8.37 (0.13)
No/yes	127 (8.3)	1.31 (1.04)	1.31 (1.06)	1.07 (1.10)	1.12 (1.11)	6.95 (0.34)	6.97 (0.35)
Yes/no	126 (8.2)	1.03 (1.05)	1.06 (1.06)	0.64 (1.11)	0.67 (1.11)	8.87 (0.34)	8.95 (0.35)
Yes/yes	378 (24.7)	1.50 (1.03)	1.50 (1.03)	1.08 (1.06)	1.09 (1.06)	6.72 (0.20)	6.63 (0.20)

% total fat mass in the highest tertile at 18 and 22 years		*p < 0.001*	*p < 0.001*	*p < 0.001*	*p < 0.001*	*p < 0.001*	*p < 0.001*
No/no	890 (58.4)	1.09 (1.02)	1.13 (1.02)	0.55 (1.04)	0.54 (1.04)	8.21 (0.13)	8.21 (0.13)
No/yes	125 (8.2)	1.23 (1.05)	1.70 (1.05)	0.98 (1.10)	1.01 (1.11)	6.65 (0.34)	6.67 (0.35)
Yes/no	152 (9.9)	1.08 (1.05)	1.24 (1.05)	0.59 (1.10)	0.61 (1.10)	9.27 (0.31)	9.34 (0.32)
Yes/yes	358 (23.5)	1.54 (1.03)	2.11 (1.03)	1.22 (1.06)	1.25 (1.06)	6.94 (0.20)	6.81 (0.21)

% trunk fat mass in the highest tertile at 18 and 22 years		*p < 0.001*	*p < 0.001*	*p < 0.001*	*p < 0.001*	*p < 0.001*	*p < 0.001*
No/no	791 (57.5)	1.07 (1.02)	1.06 (1.02)	0.54 (1.04)	0.53 (1.04)	8.28 (0.14)	8.28 (0.14)
No/yes	129 (9.4)	1.24 (1.04)	1.25 (1.06)	1.00 (1.10)	1.01 (1.11)	6.79 (0.34)	6.69 (0.35)
Yes/no	134 (9.7)	1.02 (1.06)	1.05 (1.06)	0.57 (1.11)	0.59 (1.11)	8.72 (0.34)	8.78 (0.35)
Yes/yes	322 (23.4)	1.45 (1.03)	1.47 (1.04)	1.11 (1.06)	1.14 (1.07)	6.98 (0.22)	6.86 (0.22)

Regressions performed with IL-6 and CRP on logarithmic scale – results presented in exponential means P-value by the Wald’s test for heterogeneity.

Adjusted for skin color, schooling, asset index, smoking status, harmful alcohol use, physical activity (minutes per week) and medical diagnosis of asthma, diabetes and hypertension.

**Table 4b:** Crude and adjusted linear regressions between nutritional status/body composition from 18 to 22 years and IL-6, CRP and adiponectin at 22 years, females.

	Prevalence N (%)	IL-6 (pg/mL) N = 1598 Mean (SE)		CRP (mg/L) N = 1816 Mean (SE)		Adiponectin (µg/mL) N = 1822 Mean (SE)	
		Crude	Adjusted	Crude	Adjusted	Crude	Adjusted
BMI ≥ 30 kg/m^2^ at 18 and 22 years		*p < 0.001*	*p < 0.001*	*p < 0.001*	*p < 0.001*	*p < 0.001*	*p < 0.001*
No/no	1273 (80.1)	1.19 (1.01)	1.20 (1.02)	1.29 (1.03)	1.27 (1.04)	11.23 (0.13)	11.16 (0.13)
No/yes	146 (9.2)	2.43 (1.04)	2.37 (1.05)	4.04 (1.07)	3.94 (1.10)	8.15 (0.32)	8.36 (0.34)
Yes/no	20 (1.3)	1.55 (1.11)	1.50 (1.15)	1.69 (1.21)	1.68 (1.32)	11.53 (1.01)	11.69 (1.01)
Yes/yes	150 (9.4)	2.58 (1.04)	2.49 (1.05)	3.71 (1.07)	3.75 (1.11)	8.36 (0.36)	8.65 (0.37)

Waist circumference in the highest tertile at 18 and 22 years		*p < 0.001*	*p < 0.001*	*p < 0.001*	*p < 0.001*	*p < 0.001*	*p < 0.001*
No/no	959 (60.2)	1.12 (1.02)	1.13 (1.02)	1.16 (1.04)	1.14 (1.04)	11.69 (0.14)	11.61 (0.14)
No/yes	119 (7.5)	1.75 (1.05)	1.73 (1.06)	2.50 (1.12)	2.55 (1.12)	8.63 (0.40)	8.75 (0.41)
Yes/no	110 (6.9)	1.15 (1.06)	1.12 (1.06)	1.25 (1.14)	1.19 (1.13)	11.77 (0.42)	11.71 (0.42)
Yes/yes	404 (25.4)	2.15 (1.03)	2.11 (1.03)	3.01 (1.06)	3.08 (1.06)	8.52 (0.22)	8.69 (0.22)

% total fat mass in the highest tertile at 18 and 22 years		*p < 0.001*	*p < 0.001*	*p < 0.001*	*p < 0.001*	*p < 0.001*	*p < 0.001*
No/no	935 (58.9)	1.12 (1.02)	1.09 (1.02)	1.09 (1.04)	1.09 (1.04)	11.36 (0.15)	11.32 (0.15)
No/yes	146 (9.2)	1.72 (1.05)	1.24 (1.06)	2.50 (1.10)	2.50 (1.11)	8.94 (0.37)	8.96 (0.38)
Yes/no	133 (8.4)	1.23 (1.05)	1.11 (1.06)	1.51 (1.12)	1.50 (1.11)	12.06 (0.39)	11.95 (0.39)
Yes/yes	374 (23.6)	2.15 (1.03)	1.56 (1.04)	3.31 (1.06)	3.30 (1.07)	9.12 (0.23)	9.26 (0.23)

% trunk fat mass in the highest tertile at 18 and 22 years		*p < 0.001*	*p < 0.001*	*p < 0.001*	*p < 0.001*	*p < 0.001*	*p < 0.001*
No/no	864 (58.4)	1.11 (1.02)	1.11 (1.02)	1.10 (1.04)	1.09 (1.04)	11.40 (0.15)	11.35 (0.15)
No/yes	136 (9.2)	1.71 (1.05)	1.68 (1.05)	2.28 (1.10)	2.26 (1.11)	9.03 (0.39)	9.14 (0.39)
Yes/no	133 (9.0)	1.25 (1.06)	1.27 (1.05)	1.33 (1.12)	1.31 (1.11)	12.06 (0.39)	11.91 (0.39)
Yes/yes	347 (23.5)	2.09 (1.04)	2.07 (1.03)	3.24 (1.06)	3.26 (1.07)	9.01 (0.24)	9.11 (0.24)

Regressions performed with IL-6 and CRP on logarithmic scale – results presented in exponential means P-value by the Wald’s test for heterogeneity.

Adjusted for skin color, schooling, asset index, smoking status, harmful alcohol use, physical activity (minutes per week) and medical diagnosis of asthma, diabetes and hypertension.

The same pattern was observed using different adiposity measures than BMI like WC, % of fat mass and % of trunk fat mass, however with lower magnitude compared to BMI. Around a quarter of the individuals was in the highest tertile in both evaluations of each adiposity measures (Table 4a, Table 4b).

## 4 Discussion

We found a strong and direct association in a cross sectional and longitudinal analysis, even after adjustment for confounders, between the mean IL-6 and CRP and all different measures of adiposity in the 1993 Birth Cohort from Southern Brazil, followed from birth to 22 years old. The highest increase in the mean IL-6 and CRP was for those subjects who had been in the obese category or in the highest tertile of WC and % of fat mass or for those who became obese or migrated to the highest fat mass category from 18 to 22 years. Adiponectin, on the contrary, showed an inverse association with all adiposity measurements.

Most of the literature has found this association in other countries or in a different range of age; in Latin America, however, there is scarce evidence on this and due to the epidemic of obesity in Brazil, the evaluation of such an association has been shown to be relevant.

A systematic review on ultrasensitive C-reactive protein (CRP) and anthropometric measures such as BMI and WC in Latin America has been published in 2012 [32]; eight studies met the inclusion criteria, being five from Brazil; one of the Brazilian studies was carried out in the 1982 Birth Cohort carried out in the same city as the cohort evaluated in the present paper. All these studies have found a direct association between CRP and higher BMI and WC, but they have been conducted in very small samples (except the 1982 cohort from Brazil) [33], and therefore some of them are not representative of the general population; the other limitation for these LA studies is the measure of adiposity that has been based only on BMI, WC and waist-to-hip ratio in most studies.

There is evidence in the literature that increased levels of IL-6 and CRP predict the onset of poor health outcomes, particularly cardiovascular diseases and mortality [34], [35]; it has been also found that despite the popularity of CRP, IL-6 seems to have a more robust association with age-related cardiovascular disease than CRP or fibrinogen [36].

The 1993 Birth Cohort provides an important source for evaluating the association of biomarkers and adiposity, since one of its main focus is on body composition. At the 18 and 22 follow-up visits [25], [26], not only the most common anthropometric measures were applied like BMI and WC, but also sophisticated measures of fat mass such as BOD POD and DXA. BMI reflects obesity (as a whole) without differentiating fat mass from lean mass. Waist circumference is usually recommended as measure of central obesity, mainly in young adults [37] and it could be a stronger predictor of mortality and comorbidities in adult life than BMI, but also does not differ the fat from the lean mass.

IL-6 and CRP are proinflammatory cytokine secreted by numerous tissues, like the adipose tissues [38] and higher levels have been found in obese compared with non-obese individuals and a positive correlation with fat mass [39] in most of the studies has also been found. Chronic exposure to elevated IL-6 and CRP levels is associated with the development of insulin resistance [40], the metabolic syndrome, and Type 2 Diabetes [41]. This reinforces the need for further studies on IL-6 and CRP, since they can be a potential marker of obese-related diseases.

Several covariates such as socioeconomic status, smoking, physical activity, alcohol and morbidities could play an important role in the association of adiposity and IL-6, CRP and adiponectin; the adjusted analysis in the present paper took into account these potential confounders and the results did not change after the adjustment for these confounders.

In most studies, an inverse association of low socioeconomic status (SES) and adiposity and inflammation has been shown [42], [43]. In a random sample of Mexican-American women (mean age 49.7 years), SES was inversely associated with all inflammatory markers in a cross sectional analysis, however the relationship with IL-6 was least robust, with the strongest observed socioeconomic gradient for CRP [44]. Higher adult serum concentrations of IL-6 was associated with lower SES in early childhood among mid-life adults in USA [45].

In a subsample of the Cardiovascular Health in Children III study (CHIC III) [46] it was found that higher levels of VO_2_max (maximal oxygen consumption) were associated with lower levels of IL-6, independent of obesity; we did not measure aerobic fitness in the present cohort, but we collected information on physical activity and the results from the crude to the adjusted analysis did not change after the inclusion of this covariate.

The same happened for the inclusion of alcohol and smoking as potential confounders in the analysis; although it has been mentioned in the literature that alcohol [47] and smoking [48], [49] have been directly associated with IL-6 and CRP, the present analysis did not show a relevant change in the mean IL-6 and CRP after the inclusion of these confounders.

Asthma diagnosis could also affect the association between adiposity and IL-6 and CRP since it is an inflammatory disease and there is evidence that asthma is associated with IL-6 and CRP [50], [51], but the inclusion of the covariate “medical diagnosis of asthma” did not affect the findings in the present paper.

A systematic review and meta-analysis of prospective studies investigating adiponectin and the risk for incident coronary heart disease (CHD) or stroke comprising 23,919 patients concluded that plasma adiponectin is not related to the risk for incident CHD or stroke [10]. Other systematic review and meta-analysis with a total of 24 prospective studies found that higher circulating adiponectin levels may be associated with an increased risk of CHD recurrence and all-cause/CVD mortality [52].

In other Birth Cohort from the city of Pelotas (The 1982 Cohort), a Mendelian randomization study on the causal effect of adiponectin and cardiovascular risk has been undertaken and the authors found no consistent evidence that genetic predisposition to elevated blood adiponectin levels was associated to reduced risk of cardiovascular disease [53]. Although the conflicting results on the role of adiponectin in the cardiovascular pathway, it has been demonstrated in most of the literature that adiponectin is lower among obese subjects [10], [11].

Some limitations might have limited the extent of our inferences. We cannot ascertain that individuals maintained the same levels of BMI, WC and % fat between the age of 18 and 22 years. Other biomarkers might have been of value on the pathway adiposity and inflammation like TNF-alpha and fibrinogen, besides the ones evaluated here.

## 5 Conclusions

The present study reports a prospective and cumulative association as well a contemporary association between adiposity measures and IL-6 and CRP, and an inverse association with adiponectin at 22 years in a large Birth Cohort from Southern Brazil. The fact that that we found the same direction of the association between obesity/adiposity with IL-6 as well with CRP reinforces our findings in this cohort; moreover, the inverse association between adiposity with adiponectin also shows the consistency of this study. Due to the increasing prevalence of the obese-related diseases worldwide it becomes imperative to identify clinically useful measures of inflammation in order to better predict risk for morbidity and mortality.

## 6 Funding

This work was supported by the Science and Technology Department, Brazilian Ministry of Health, with resources transferred through the Brazilian National Council for Scientific and Technological Development (CNPq) [grant number 400943/2013-1]. The study ‘Pelotas Birth Cohort, 1993’ was conducted by the Postgraduate Program in Epidemiology at Universidade Federal de Pelotas, with the collaboration of the Brazilian Public Health Association (ABRASCO). From 2004 to 2013, the Wellcome Trust supported the 1993 Birth Cohort study [grant number 086974/Z/08/Z]. The initial phases of the cohort were funded by the European Union and the Brazilian National Program for Centers of Excellence (PRONEX), the National Research Council (CNPq), and the Ministry of Health.
